# Lawson on Cholera

**Published:** 1849-01

**Authors:** 


### LAWSON ON CHOLERA.

We received a few days since, a pamphlet of 58 pages, on the Nature and Treatment of Cholera, from the pen of Dr. L. M. Lawson, Professor of Materia Medica and Therapeutics and General Pathology in the Medical College of Ohio. The work is reprinted from the *Western Lancet,* and embraces a historical sketch of the disease, the causes which produce it, its mode of propagation, its pathology, prevention and medical treatment. It is a seasonable publication on a subject which is exciting anxious inquiry in all classes of the community, and concerning which physicians are expected to be maturing their views anticipative of the appearance of the epidemic. Dr. Lawson presents here a faithful digest of professional experience and observation concerning cholera, and we heartily recommend his carefully prepared work to our readers.

